# Challenges in diabetes mellitus type 2 management in Nepal: a literature review

**DOI:** 10.3402/gha.v9.31704

**Published:** 2016-10-18

**Authors:** Bishal Gyawali, Alessandra Ferrario, Edwin van Teijlingen, Per Kallestrup

**Affiliations:** 1 Center for Global Health, Department of Public Health, Aarhus University, Aarhus, Denmark; 2 Nepal Development Society (NEDS), Bharatpur, Nepal; 3 LSE Health, London School of Economics and Political Science, London, United Kingdom; 4 Faculty of Health & Social Sciences, Bournemouth University, Dorset, United Kingdom

**Keywords:** diabetes mellitus type 2, diabetes complications, costs, low-income country, health care, Nepal

## Abstract

**Background and objectives:**

Diabetes has become an increasingly prevalent and severe public health problem in Nepal. The Nepalese health system is struggling to deliver comprehensive, quality treatment and services for diabetes at all levels of health care. This study aims to review evidence on the prevalence, cost and treatment of diabetes mellitus type 2 and its complications in Nepal and to critically assess the challenges to be addressed to contain the epidemic and its negative economic impact.

**Design:**

A comprehensive review of available evidence and data sources on prevalence, risk factors, cost, complications, treatment, and management of diabetes mellitus type 2 in Nepal was conducted through an online database search for articles published in English between January 2000 and November 2015. Additionally, we performed a manual search of articles and reference lists of published articles for additional references.

**Results:**

Diabetes mellitus type 2 is emerging as a major health care problem in Nepal, with rising prevalence and its complications especially in urban populations. Several challenges in diabetes management were identified, including high cost of treatment, limited health care facilities, and lack of disease awareness among patients. No specific guideline was identified for the prevention and treatment of diabetes in Nepal.

**Conclusions:**

We conclude that a comprehensive national effort is needed to stem the tide of the growing burden of diabetes mellitus type 2 and its complications in Nepal. The government should develop a comprehensive plan to tackle diabetes and other non-communicable diseases supported by appropriate health infrastructure and funding.

## Introduction

Cardiovascular disease, cancer, diabetes, chronic respiratory diseases, and other non-communicable diseases (NCDs) kill more than 36 million people each year and they are responsible for nearly half of the global burden of disease (1). In particular, these NCDs are of increasing concern in low- and middle-income countries (LMICs) where 80% of all global NCD deaths occur. The World Health Organization (WHO) projects that the burden of NCDs will increase rapidly predicting NCD deaths to increase by 15% globally between 2010 and 2020 (2).

This rising prevalence of NCDs is associated with increasing urbanization, the influence of globalization on consumption patterns, and an aging population (3). Importantly, many LMICs will face higher levels of NCDs at earlier stages of their economic development, with fewer resources, and with less time to respond effectively (4). People in LMICs tend to develop disease at younger ages, suffer longer, and die sooner compared to high-income countries, which can undermine the economic development of these countries. Diabetes is emerging as a major global problem worldwide and is reaching epidemic proportions with global prevalence of 8.3%, affecting 387 million adults and costing 612 billion dollars in health care spending in 2014 (5).

In 2013 Nepal had one of the lowest gross domestic products (GDPs) per capita at US$ 1,500 (6). Its economy is characterized by low productivity and growth, making the country heavily dependent on external aid. The total population of Nepal is nearly 27 million with an annual growth rate of 1.6% (7), of which 17% live in cities. Currently, Nepal is the fastest urbanizing country in South Asia (4.9% per year) (8). Poverty remains a serious problem with about a quarter living below the poverty level of US$ 1.25 per day (9). Although the proportion of people living in poverty has declined from 42 to 25% over the past 15 years, it still remains high in the rural settings, where poverty levels are between 1.8 and 10 times higher than in the cities (10). Life expectancy in Nepal has increased steadily over the past 20 years to 67.9 years for males and 65.5 years for females (11).

Nepal is experiencing an epidemiological transition with a prevalence of NCDs estimated to range from 31% (12) to 36.5% (13). The four most prevalent NCDs are chronic obstructive pulmonary disease (COPD), cardiovascular disease, diabetes, and cancer (12). NCDs were estimated to account for 60% of all deaths in Nepal in 2014 (14). The projected increase in the burden of NCDs is largely driven by the globalization and urbanization. These determinants in turn contribute to the common chronic disease risk factors of unhealthy diet, inactive lifestyle, harmful use of alcohol, and tobacco use and finally increase the chance of developing hypertension, obesity, and diabetes.

A recent systematic review and meta-analysis showed a prevalence rate of diabetes mellitus type 2 (DMT2) of 8.4% in Nepal (15). Relative to neighbouring countries such as Pakistan, Sri Lanka, and Bangladesh, Nepal has a higher prevalence of DMT2 and impaired glucose tolerance (16). However, this finding about Nepal should be handled with caution as no large-scale studies have been conducted.

The health care system in Nepal is facing several challenges. Most health care facilities are concentrated in urban areas while rural health facilities often lack resources, staff, and infrastructure (17). The country has one of the world's most deprived health systems, with a density of medical doctors, nurses, and midwives of 0.67 per 1,000 population, far less than the WHO's benchmark of 2.3 health care professionals per 1,000 population (18). Nepal's health care deficits are largely attributed to low government spending, unevenly distributed health services, limited affordability, inadequate supply of essential drugs, low awareness of disease and possible treatment, and poor retention of human resources in rural areas (19). Nepal spends only 6% of its GDP on health care (20). Of this, most of the expenditure (about 70%) is private out-of-pocket (21). In view of this high share of out-of-pocket expenditure on health care, the Government of Nepal is working towards universal health coverage in line with the Sustainable Development Goals (SDGs) to provide: 1) universal access to basic health services, and 2) free essential drugs and diagnostics as part of the New National Health Policy (22). Nepal initiated a free essential health care services (EHCS) programme in 2007, which included a limited range of free care services in the publicly funded primary health care and secondary health care with a limited number of free essential medicines (23).

Treatment and management of diabetes is a major challenge in Nepal, for reasons such as low disease awareness among the population; various socio-cultural factors; educational strategies; and paucity of programmes to detect, manage, and prevent diabetes and its complications (15). Health care professionals and policymakers have to come together to assess the increasing burden of diabetes and design appropriate preventive and management strategies. This study aims to review evidence on the prevalence of DMT2 and its complications, the cost, treatment, and challenges to be addressed to contain the epidemic and its negative economic consequences. To our knowledge, no such study has been carried out. This literature review critically assesses the evidence, and will make recommendations to help improve diabetes management in Nepal.

## Methods

A review of available evidence was conducted on PubMed on DMT2 and complications, its associated cost, expenditure, treatment, and prevention in Nepal. We performed a manual search for other articles and additional references from published articles through Google. We also included reports and articles from the WHO (24, 25), the International Diabetes Federation (IDF) (16), the Government of Nepal (26–28), and a conference presentation (29).

A search of the online databases for studies published in English was performed using the keywords: ((‘diabetes’ [Title/Abstract] AND ‘NEPAL’ [Title/Abstract])) OR ((‘Diabetes Mellitus’ [Mesh] OR ‘Diabetes Mellitus, Type 2’ [Mesh] AND ‘NEPAL’ [Mesh])) in PubMed. To ensure the most current and relevant studies, the search was limited to studies published between January 2000 and November 2015. The search was conducted in December 2015. Studies presenting evidence on prevalence, costs, complications, treatment, and prevention were included in the analysis. Studies published in languages other than English were excluded. We also consulted with national NCD experts during literature gathering. If costs data were reported only in Nepalese Rupee (NPR), we converted the amounts into United States dollar (US$).

## Results

**Fig. 1:**
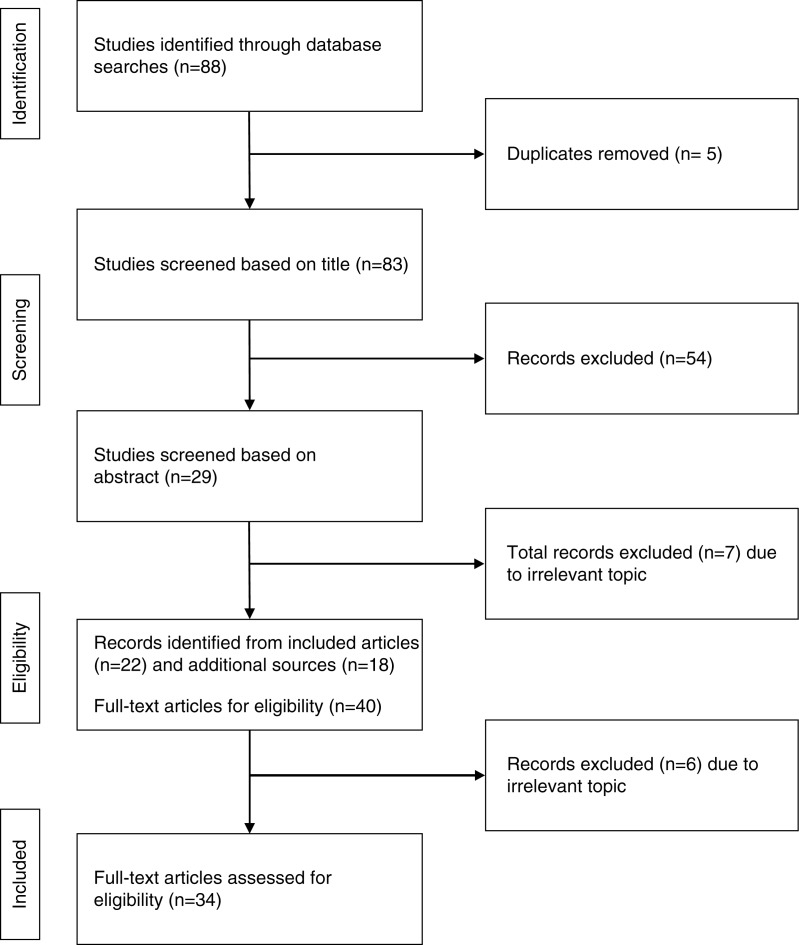
Flow chart of the study selection process.

The search strategy yielded 88 papers in PubMed. Then duplicates (*n*=5) and irrelevant papers based on title (*n*=54) were excluded and seven were excluded after reading the abstract. A further five additional peer-reviewed papers were identified through Google and seven were identified through reference searching including grey literature and presentations, leaving a total of 34 papers for analysis (Fig. 1). International studies, national surveys, and small cross-sectional surveys on DMT2 in Nepal were included (Table 1). Table 2 summarizes the relevant literature on DMT2.

**Table 1:** List of articles included in the analysis

Author	Title	Publication year	Reference
Baral et al.	Evaluation of new WHO diagnostic criteria for diabetes on the prevalence of abnormal glucose tolerance in a heterogeneous Nepali population – the implications of measuring glycated hemoglobin	2000	(30)
Karki et al.	Prevalence of non-insulin dependent diabetes mellitus in urban areas of eastern Nepal: a hospital based study	2000	(31)
Singh and Bhattarai	High prevalence of diabetes and impaired fasting glycaemia in urban Nepal	2003	(32)
Jha	Cost analysis for management of type-2 diabetes: a case study of rural and urban setting	2004	(33)
Sasaki et al.	The prevalence of diabetes mellitus and impaired fasting glucose/glycaemia (IFG) in suburban and rural Nepal-the communities-based cross-sectional study during the democratic movements in 1990	2005	(34)
Shrestha et al.	The prevalence of hypertension and diabetes defined by fasting and 2-h plasma glucose criteria in urban Nepal	2006	(35)
Mehta et al.	Risk factors, associated health problems, reasons for admission and knowledge profile of diabetes patients admitted in BPKIHS	2006	(36)
Ono et al.	The prevalence of type 2 diabetes mellitus and impaired fasting glucose in semi-urban population of Nepal	2007	(37)
Kart et al.	Lay explanations and self-management of diabetes in Kathmandu, Nepal	2007	(38)
Upadhyay et al.	Prescribing pattern in diabetic outpatients in a tertiary care teaching hospital in Nepal	2007	(39)
Shrestha et al.	Prevalence of and factors associated with diabetic retinopathy among diabetics in Nepal: a hospital based study	2007	(40)
Upadhyay et al.	Knowledge, attitude and practice about diabetes among diabetes patients in western Nepal	2008	(41)
Paudyal et al.	Prevalence of diabetic retinopathy following a community screening for diabetes	2008	(42)
Chettri and Chapman	Prevalence and determinants of diabetes among the elderly population in the Kathmandu Valley of Nepal	2009	(43)
Rajbhandari	Diabetes in Nepal-future and perspective	2010	(29)
Mehta et al.	Hyperglycemia, glucose intolerance, hypertension and socioeconomic position in eastern Nepal	2011	(36)
Sharma et al.	Prevalence of hypertension, obesity, diabetes, and metabolic syndrome in Nepal	2011	(44)
Thapa et al.	Demographics and awareness of diabetic retinopathy among diabetic patients attending the vitreo-retinal service at a tertiary eye care center in Nepal	2012	(45)
International Diabetes Federation	IDF Diabetes Atlas: country estimates table 2011	2012	(16)
Sharma et al.	Community-based screening for chronic kidney disease, hypertension and diabetes in Dharan	2013	(46)
Shrestha et al.	Cost of diabetes mellitus care among patients attending selected outpatient clinics	2013	(47)
Government of Nepal	Multisectoral action plan for the prevention and control of non-communicable diseases (2014–2020)	2013	(26)
Prasai	A review of studies on Nepal's national free health care programme	2013	(28)
Parajuli et al.	Factors associated with nonadherence to diet and physical activity among Nepalese type 2 diabetes patients; a cross sectional study	2014	(48)
Saito et al.	Catastrophic household expenditure on health in Nepal: a cross-sectional survey	2014	(25)
Aryal et al.	Non communicable diseases risk factors: STEPS Survey Nepal 2013	2014	(24)
Poudel	Government expands essential drugs list, focus shifts to non-communicable diseases	2014	(49)
Poudel	Diabetes and endocrinology in Nepal	2014	(50)
Gautam et al.	Diabetes related health knowledge, attitude and practice among diabetic patients in Nepal	2015	(51)
Maskey et al.	Hypothyroidism in diabetes mellitus patients in Eastern Nepal	2015	(52)
Mishra et al.	Depression and health-related quality of life among patients with type 2 diabetes mellitus: a cross-sectional study in Nepal	2015	(53)
Joshi et al.	Illness perception and depressive symptoms among persons with type 2 diabetes mellitus: an analytical cross-sectional study in clinical Settings in Nepal	2015	(54)
Aryal et al.	The burden and determinants of non communicable diseases risk factors in Nepal: findings from a nationwide STEPS survey	2015	(27)
Kalra et al.	Place of sulfonylureas in the management of type 2 diabetes mellitus in South Asia: A consensus statement	2015	(55)

**Table 2:** Relevant articles on various areas of DMT2

Areas of diabetes management	Number of papers	References
Prevalence	12	(16, 24, 27, 30–32, 34, 35–37, 44, 46)
Risk factors	7	(31, 35, 36, 38, 43, 44, 51)
Complications	9	(35, 36, 39, 40, 42, 45, 52–54)
Cost	4	(25, 33, 39, 47)
Treatment	5	(26, 33, 41, 49, 55)
Challenges in diabetes management	7	(28, 29, 36, 41, 48, 50, 51)

### Prevalence of DMT2 and its complications

#### Prevalence

The IDF estimates that the prevalence of DMT2 in Nepal was 4.5% in 2012 and the predicted number of undiagnosed cases in adults was 294 per 1,000 population (16). Various cross-sectional studies reported prevalence of DMT2 in different settings. A study by Shrestha et al. in 2006 reported a prevalence of 19% of DMT2 in urban Nepal (35). This study also reported that 54.4% (53.8% of men and 55.1% of women) were undiagnosed (35), similar to rates reported elsewhere (32, 36, 37). Compared to rural prevalence, the urban one is high (14.6% of DMT2 among city dwellers in 2003) (32). This study reported a rural prevalence of 2.5%. Yet in a rural study in Nepal, Sasaki et al. reported a much lower prevalence of 0.3% in 2004 (34). Studies reporting prevalence in rural areas show consistently lower prevalence estimates than those studies reporting urban prevalence. Caution is however needed when interpreting the results due to different methodologies applied.

#### Risk factors

Studies reported non-modifiable risk factors for DMT2 including increased age (31, 35, 44), being a woman (32, 35, 36, 44) and altered immunity (38) and several modifiable risk factors such as urban residency (36), higher socio-economic status (36), higher body mass index (BMI) (36), lack of physical activity (44, 51), low education (36), hypertension (35, 36), and disturbed sleep and family history of hypertension (43). A study to explore lay explanations and self-management of diabetes in urban Nepal reported stress or worry, diet or eating habits, heredity, pollution in the environment, and self-care behaviour as the main causes of DMT2 (38), while another institution-based cross-sectional study reported alcohol and tobacco use as common risk factors for DMT2 (51).

#### Diabetes complications

Diabetes can lead to serious complications and is associated with a range of comorbidities. The literature suggests that the prevalence of complications and comorbid diseases in DMT2 patients were retinopathy (19.3–78%), hypertension (36.7–70.6%), other ocular problems (39%), renal problems (25%), neurological problems (25%), diabetic foot (21.4%), depressive symptoms (6.2–54.1%), gastritis (8.5%), angina (5.1%), and hypothyroidism (4.1%) (Table 3). Evidence shows that diabetes complications can significantly inflate the cost of diabetes in Nepal. It is reported that the total direct cost per year for a patient living with diabetes for 16–20 years of illness was approximately 161% higher than for a patient with a history of DMT2 for 1–5 years (47).

**Table 3:** Diabetes-related complications

Estimates	Study year	Sample frame and sample	Study design	Diagnostics	Reference
Diabetic retinopathy					
19.3%	2008	Sample frame: Semi-urban setting Sample: 34 DMT2 patients Mean age: 54.7±12 years	Community-based cross-sectional study	Blood samples, ocular examination	(42)
44.7%	2005–2006	Sample frame: Tertiary eye care centre Sample: 371 DMT2 patients Mean age: 57.4±12 years	Hospital-based cross-sectional study	Blood samples, ocular examination	(40)
78%	2005–2006	Sample frame: Tertiary eye care centre Sample: 210 DMT2 patients Mean age: 57±10.4 years	Hospital-based cross-sectional study	Blood samples, ocular examination	(45)
Hypertension					
70.6%	2006	Sample frame: outpatient pharmacy (OPP); urban setting Sample: 182 DMT2 patients Mean age: 56.9±12.6 years	Cross-sectional study	NR	(39)
60.7%	2003–2004	Sample frame: Medical units Sample: 310 DMT2 patients 60% women, 40% men	Hospital-based exploratory study	NR	(36)
36.7%	2001–2002	Sample frame: Seven wards of metropolitan and sub-metropolitan municipalities Sample: 105 DMT2 patients 13.2% women, 11.5% men	Field survey	Fasting and 2 h plasma glucose, Blood Pressure measurement	(35)
Renal problem					
25%	2003–2004	Sample frame: Medical units Sample: 310 DMT2 patients 60% women, 40% men	Hospital-based exploratory study	NR	(36)
Neurological problem					
25%	2003–2004	Sample frame: Medical units Sample: 310 DMT2 patients 60% women, 40% men	Hospital-based exploratory study	NR	(36)
Other ocular problem					
39%	2003–2004	Sample frame: Medical units Sample: 310 DMT2 patients 60% women, 40% men	Hospital-based exploratory study	NR	(36)
Diabetes foot					
21.4%	2003–2004	Sample frame: Medical units Sample: 310 DMT2 patients 60% women, 40% men	Hospital-based exploratory study	NR	(36)
Hypothyroidism					
4.1%	2012–2013	Sample frame: Urban setting Sample: 271 DMT2 patients	Hospital-based descriptive study	Thyroid function test	(52)
Depressive symptom					
54.1%	2014	Sample frame: Tertiary hospital Sample: 157 DMT2 patients 60.5% women, 39.5% men	Cross-sectional surveys	PHQ-9 scale	(53)
44.1%	2013–2014	Sample frame: Tertiary hospital Sample: 379 DMT2 patients Mean age: 54.8±10.7 years	Analytical cross-sectional study	BDI-II scale	(54)
6.2%	2006	Sample frame: OPP; urban setting Sample: 182 DMT2 patients Mean age: 56.9±12.6	Cross-sectional study	NR	(39)
Gastritis					
8.5%	2006	Sample frame: OPP; urban setting Sample: 182 DMT2 patients Mean age: 56.9±12.6	Cross-sectional study	NR	(39)
Angina					
5.1%	2006	Sample frame: OPP; urban setting Sample: 182 DMT2 patients Mean age: 56.9±12.6	Cross-sectional study	NR	(39)

NR=Not Reported.

#### Diabetes cost

Few hospital-based studies reported evidence on cost of diabetes in Nepal (Table 4). A study on the cost of 227 diabetic patients at four outpatient private clinics in Kathmandu was conducted in 2010 (47). The mean total cost per visit paid out-of-pocket by patients with diabetes in an outpatient clinic was reported to be US$ 13.3. The study also reported that the total cost incurred in the treatment and care of diabetes per month was US$ 40.4 and US$ 445 per annum. Medicinal costs accounted for the majority (80%) of the total direct cost per visit (US$ 11). The study concluded that diabetes patients in the private sector of Nepal experience a high cost burden.

**Table 4:** Cost of diabetes in Nepal

Source of cost	Study year	Study setting	Amount (NPR)	Amount (US dollar)	Reference
Average cost per prescription	2006	Sample frame: outpatient pharmacy (OPP); Urban setting Sample: 182 DMT2 patients Study design: cross sectional	1156.2	16.2	(39)
Total cost per visit	2010	Sample frame: A public hospital, a private hospital and two polyclinics one each in Kathmandu and Lalitpur Sample: 227 DMT2 patients Study design: cross sectional	NR	Mean cost per patient per visit Public sector: Mean 5.1, SD 253, 95% CI 4.3–5.9 Private sector: Mean 17.5, SD 948.2, CI 15.4–9.6	(47)
Total cost per month	2010	Sample frame: A public hospital, a private hospital and two polyclinics one each in Kathmandu and Lalitpur Sample: 227 DMT2 patients Study design: cross sectional	NR	Mean cost of illness per patient per month Mean 40.4, SD 2232.7, 95% CI 36.4–44.5	(47)
Total cost per annum	2010	Sample frame: a public hospital, a private hospital and two polyclinics one each in Kathmandu and Lalitpur Sample: 227 DMT2 patients Study design: cross sectional	NR	Mean cost per patient per annum Mean 445.9, SD 27534.9, 95% CI 396.1–495.6	(47)
Total cost per annum	2002–2003	Sample frame: Two sites in Dhulikhel community and Kathmandu's Diabetes clinic; rural and urban setting Sample: 60 DMT2 patients Study design: Prospective observational follow-up	Cost to the health system and the patient per annum 5470	NR	(33)

NR=Not Reported.

In LMICs, the cost of prescription drugs can make up for a substantial proportion of total cost of illness in many chronic diseases including diabetes. A 2007 study on demographic profiles and patterns of drug prescribing among ambulatory patients with diabetes at an urban outpatient pharmacy (OPP) found that the average cost per prescription per visit was US$ 16.2 (39). The study reported a very large variation in cost among trade names of particular drugs. The study found that anti-diabetic medications constituted 58.9% of the total cost of medications. Among the anti-diabetic medication, insulin was responsible for the highest proportion (41.1%) of the total cost incurred on anti-diabetics followed by biguanides (32.6%). A 2002 study, in both rural and urban Nepal, estimated the median expenditure on diabetes health care to US$ 76.5 excluding other expenses of a diabetic patient such as for home blood glucose monitor, test strips, lancets, urine sticks, blood pressure measuring equipment, and exercising machines (33). The study also concluded that diabetes is one of the most costly and burdensome NCDs on families and on the health care system.

Health expenditures (including drugs) are largely paid for out-of-pocket in Nepal. A study conducted in 2011–2012 in Kathmandu provided evidence relating diabetes illnesses to catastrophic out-of-pocket expenditure on health care (25). The study reported that more than one in every seven households experienced catastrophic expenditure on health. It concluded that injuries and major NCDs including diabetes, asthma, and heart disease were frequently associated with catastrophic spending in the poorest household.

### Treatment

#### National guidelines for diabetes prevention and treatment

Currently, there is no specific guideline for the prevention and treatment of DMT2 although the government has recently spearheaded a Multi-sectoral Action Plan on the ‘Prevention and Control of NCDs 2014–2020’ to reduce preventable morbidity, avoidable disability, and premature mortality as a result of major NCDs (26). The action plan contains broad strategic actions and key milestones to be achieved within a specified time frame such as decreasing the overall mortality of diabetes by 25% and halting the rise in obesity and diabetes by the year 2025, strengthening health system competence particularly primary health care to address major NCDs, and empowering communities and individuals to self-care. Moreover, the plan envisages the adoption of the WHO Package of Essential NCD (PEN) guidelines to screen, diagnose, treat, and refer DMT2 at village, primary health care center (PHCC), and hospital levels. The plan emphasizes on development and implementation of well-structured media campaigns outlining dose, medium and timing of information on NCDs to raise awareness on diabetes, as well as the establishment of baseline information by conducting a burden of disease study including diabetes. At the moment, the government has included medicines of 12 NCDs in the list of essential drugs that are distributed free of charge (49).

#### Access to treatment and medications

Few studies have examined access to essential drugs for diabetes in Nepal. Upadhya et al. in 2007 reported that out of 685 total drugs prescribed at the hospital, 314 (45.8%) were prescribed for treatment of diabetes. Among them, biguanides accounted for 51.3%, followed by sulfonylureas (35.4%), insulin (8.0%), thiazolidinediones (4.8%), meglitinides, and alpha glucosidase inhibitors (0.3%) (39). Metformin, being cheap (and affordable), was the most commonly prescribed drug; 56% of urban patients were treated with sulphonylureas and 46% of rural patients with metformin (33). Essential diabetes drugs such as insulin, metformin, glibenclamide, protamine zinc insulin, and glipizide are included in the national drug list to which all publicly insured patients have access (55).

### Challenges in diabetes management

#### Knowledge, attitude, and practice towards diabetes

Patients’ lack of knowledge and attitude towards diabetes care can hinder their ability to manage their disease. Published studies on DMT2 knowledge, attitude, and practice (KAP) showed that there is a poor level of KAP about diabetes in Nepal. Attitude was defined as the perception of responsibility towards the disease, and practice was measured by assessing perception of adherence to diabetes self-care recommendations. A 2015 study of 244 diabetes patients in urban Nepal reported that 21.3% had highly insufficient knowledge, 22.5% had insufficient knowledge of diabetes, and 28.3% had poor attitudes (51). Similarly, the level of practice score was also poor at 29.1%. This shows that the potential diabetes-related literacy was low. Another study conducted in 2014 in a tertiary-level care hospital of urban Nepal showed that half of all 385 DMT2 patients had poor knowledge level about diabetes (48). Upadhya et al. noted a lack of awareness of diabetes even in patients attending hospital in western Nepal who had had the disease for a long time (41). Likewise, a hospital-based study in 2006 found that only few patients with diabetes had good knowledge of causes, curability, treatment modalities, diet, and other aspects of DMT2 (36).

#### Coverage of services

Nepal has a pluralistic health system with different health care facilities, including public and private (56). Public or the government health facilities have a network of sub-health posts, health posts, PHCCs, and district hospitals. Although free health care services are available through government health facilities, the national priority in health care is still the prevention and control of communicable diseases, and maternal and child health services (19). This means the current health care system is not optimally equipped to deal with the double burden of dealing with the ‘old’ low-income country diseases as well as the new challenges brought on by diabetes and other NCDs. Diabetes management requires long-term follow-up with continuous access to medication and specialist care; however, diabetes care is not a priority for many health care staff. There is no routine measurement of blood glucose level as most of the reliable government-owned laboratories are located in the capital city (50). PHCCs often lack blood testing equipment, essential drugs, and diabetes specialists. One study found expired drugs in district hospitals and PHCCs especially in mountain districts as well as these facilities running out of supplies all together (28). Private health facilities, on the other hand, operate in a largely unregulated market (57). The private share of total health expenditure in Nepal is 70%, of which about 85% comes from out-of-pocket payments, indicating a significant involvement of private facilities in health provision (58). It is clear that health care costs put a heavy burden on the poor jeopardizing their access to the health system, as they cannot afford private health care.

Moreover, management of DMT2 and its complications is a formidable challenge to the government owing to lack of appropriate infrastructure and financing scheme required to provide diabetes management services, and inadequate essential drugs coverage (28). There is a lack of government support or subsidy that might result in unaffordable costs. There is inconsistent practice and limited consultations concerning diabetes diagnosis and management among health care providers in Nepal (50). The health system is further moderated by lack of access to a complete multidisciplinary diabetes health care system, lack of centralized data system, lack of quality scientific research in diabetes, and lack of post-graduate courses in diabetes (29). Although primary health care is the backbone of health service delivery, this level of health care does not have preventive and curative services for NCDs. At present, the majority of NCDs services are provided by tertiary-level specialist health institutions, non-government organizations (NGOs), and private sectors, most of which are clustered in urban areas. Organizations such as Nepal Diabetes Association (59) and Astha Nepal (60) are currently organizing public awareness campaigns on diabetes management through free health camps, while another national non-profit network, the Nepal Diabetic Society, is currently working to provide diabetic care to patients (61).

To date, there is still no national screening and prevention programme for diabetes albeit the government has launched the aforementioned Multi-sectoral Action Plan for the prevention and control of NCDs. Moreover, the most common diagnostic methods used are based on glucose criteria such as fasting, postprandial, and random blood glucose levels (29). Recently, the HbA1c test has been endorsed as a diagnostic test in high-income countries, as a superior alternative to glucose-based criteria. There is still controversy about implementation of HbA1c criteria in Nepal due to lack of standardization of the HbA1c measurement methodology and high cost. There are only a few reliable laboratories for glucose measurement and most of these are in the capital and affected by lack of quality control (50).

## Discussion

This systematic review is the first comprehensive analysis on prevalence, risk factors, cost, complications, treatment, and management of diabetes in Nepal to date. Our results show that the country is facing an increasing prevalence of DMT2 and its complications. Moreover, there is marked countrywide variation in DMT2 prevalence, with one study reporting nearly one in five affected in urban Nepal (35) and as low as 0.3% in rural Nepal (34).

According to the Sixth Edition of the IDF Atlas (2013), the national diabetes prevalence of Nepal was 4.5% of the adult population between 20 and 79 years of age (16). The 2015 edition of the IDF Atlas, which was published after we completed our study, updated the national estimate to 3.3% (62). The difference in prevalence estimates in different settings could be due to differences in which the analytic populations were defined. For instance, the IDF estimate takes into account both the urban and the rural populations. Furthermore, the comparison of these figures should be done with caution due to differences in methodology such as different diagnostic criteria used, different methods adopted, how representative samples are, and the varying time periods in which the studies have been performed.

In addition, there is rapid urbanization and the internal migration from rural to urban areas has contributed to the increasing rates of DMT2 (15). Despite an increased burden of disease, there has been minimal effort with regard to its prevention and control in Nepal. Meanwhile, the health care system of Nepal is faced with profound challenges including inequity in health care access, scarcity of resources, insufficient and untrained human resources, and financial sustainability (50, 63). Therefore, there is an immediate need to develop a comprehensive and integrated strategy involving planning, action, and implementation from the government and all stakeholders to address the burden of diabetes and control its modifiable risk factors. The strategic plan should focus on building and strengthening the primary health care network with an emphasis on disease screening, prevention, risk-factor control, and health promotion.

Our review found that patients’ lack of knowledge about diabetes care limiting patients’ ability to improve diabetes control through self-management (41, 48, 51). There is also lack of public awareness regarding diabetes especially in remote places where medical services are poor (31). Therefore, Nepal needs to encompass strategies to reach out to the general population to spread awareness of the disease and its complications as well as educate and reach out to diabetes patients to improve outcomes. Educational programmes must be simultaneously planned to educate and sensitize various stakeholders in diabetes management.

The economic burden of diabetes is enormous (47). Diabetes is costly because of its chronic nature, the severity of its complications, and the modalities required to control them. Consequently, people have frequent and intensive encounters with the health system such as higher use of hospital inpatient care, outpatient visits, emergency visits, and prescription drugs. Moreover, the out-of-pocket expenses associated with diabetes remain a barrier to the prevention of diabetes-related complications in Nepal. As a result, diabetic patients tend to forgo appropriate management due to unaffordable cost of the treatment (41). This calls for more innovative ways of financing health care for chronic NCDs in Nepal. There is also a great variation in cost of prescription drugs used to treat diabetes in Nepal, as found in India (64) and elsewhere (65). One tool to decrease the prescription cost is to prescribe less-expensive trade names provided that the quality of the alternative is good. People with diabetes may sometimes need to take two or three different pills at a time. In this case, combination medications – one tablet that contains two or more types of medications combined – may also offer substantially more benefits, help increasing compliance and, depending on the market price, potentially even be cost saving (66).

Studies focusing on access to diabetes care and treatment are very limited in Nepal. However, one study showed that patients’ adherence to diabetes care and treatment was higher among those who were nearer to hospital (48). Patients who were nearer had more frequent visits to the health care provider, with better follow-up than those who were far. Thus diabetes care and treatment can be affected by the availability and cost of screening tools, especially among less informed populations (38). Furthermore, in Nepal the availability of free essential medicines, including diabetes was poor in rural public hospitals (28). There was also variation in prices of medications in urban and rural areas, which might result in poor adherence to diabetes medications (33, 55). Policymakers should take a broader view that encompasses barriers to access to care and treatment. Screening of those at increased risk and treatment of symptomatic patients should be in place in all health care settings. Health systems need to guarantee equitable access to medicines and technologies, which are of assured quality, safety, efficacy, and cost-effectiveness and ensure that these are used in an evidence-based and cost-effective manner (67).

The high levels of illness associated with diabetes and other NCDs have compelled the Government of Nepal to take actions on NCDs or make them a priority. The Multi-sectoral Action Plan on the Prevention and Control of NCDs 2014–2020 (26) offers opportunities for improving care for diabetes through provisions at the primary levels of health care. The government's initiative of including NCDs drugs (68) and more recently increasing the types of NCDs drugs on the free essential drugs list (69) is one of the important steps to integration at primary health care level to deliver health services cost-effectively and efficiently. Moreover, the government has recently drafted the New National Health Sector Programme III for the period 2015–2020, which focuses on strengthening the primary care system through early treatment of NCDs, including diabetes in both urban and rural settings. Despite this, recent initiative challenges remain. The growing problem of NCDs including diabetes requires the input and influence of all sectors of societies, including local communities, who play an enormous role in bringing changes in attitudes towards health and lifestyle (70). However, there is lack of experience with community-based prevention for diabetes in the policy. Health care in general and diabetes in particular are very heavily dependent on adequate infrastructure and funding. Government funding for preventive health programmes remains too low to effectively respond to the growing burden of diabetes. Innovative financing mechanisms should be explored for generating funds for diabetes prevention and control.

Our study is limited to the selected database source and English-language literature, and this might have missed some relevant articles. Most of the available diabetes studies in Nepal were institution-based and urban-focused, leaving a rural research information gap. Moreover, our study included only those studies that were available via electronic media, which could have resulted in the exclusion of studies available in a paper-based format. Nevertheless, with consideration of these limitations, this review provides a better understanding of prevalence, cost, and treatment of DMT2 and its complications in Nepal and identifies the challenges to be addressed to contain the epidemic.

## Conclusions

DMT2 is emerging as a major health care problem in Nepal, with rising prevalence and its complications especially in urban populations. Diabetes prevalence was associated with various modifiable and non-modifiable risk factors. Nepal faces several challenges in diabetes management, including limited health care facilities, high cost of treatment, lack of disease awareness among patients, and lack of specific guidelines for the prevention and treatment of diabetes. Diabetes treatment and prevention efforts were further impeded by the country's health system that places a higher priority on communicable diseases and maternal and child health services and by a private health system focused on curative medicine. There is an urgent need to strengthen the health system so as to enable it to effectively face the challenges posed by diabetes and other NCDs. Preventive strategies must take into account the growing prevalence of risk factors associated to these diseases. Given the magnitude and complexity of the diabetes burden, there is an immediate need for a broad-based approach, and involvement of relevant stakeholders from the government, civil society, and the private sector for early detection and primary and secondary prevention of diabetes and its complications. The government should develop a comprehensive action plan to tackle diabetes and other NCDs with clear responsibilities, a monitoring plan backed by the necessary funds for public awareness about risk reduction behaviours, and availability of essential medicines to all sectors of community.

## Paper context

Diabetes mellitus type 2 has emerged as a major health care problem in Nepal. This low-income country faces several challenges in diabetes management due to a rising prevalence and its complications, cost of treatment, limited health care facilities, lack of disease awareness among patients, and lack of specific guidelines for the prevention and treatment of diabetes. The government should develop a comprehensive plan to tackle diabetes and other non-communicable diseases supported by appropriate health infrastructure and funding.
